# Effect of salinity on the feeding behavior of *Cordylophora caspia* (Pallas, 1771) (Hydrozoa)

**DOI:** 10.1093/plankt/fbaf065

**Published:** 2026-01-07

**Authors:** S Nandini, S S S Sarma, Henri J Dumont

**Affiliations:** Laboratory of Aquatic Zoology, UMF, FES Iztacala, Universidad Nacional Autónoma de México, Av. de Los Barrios #1, Los Reyes, Ixtacala, Tlalnepantla Postal Code 54090, Mexico; Laboratory of Aquatic Zoology, UMF, FES Iztacala, Universidad Nacional Autónoma de México, Av. de Los Barrios #1, Los Reyes, Ixtacala, Tlalnepantla Postal Code 54090, Mexico; Ghent University, Faculty of Sciences, Department of Biology, KL Ledeganckstraat 35, 9000, Belgium

**Keywords:** Cnidaria, copepods, functional response, prey preference, rotifers

## Abstract

*Cordylophora caspia,* is considered an invasive species in America. We isolated this hydrozoan from the brackish water section of the River Tuxpan (State of Veracruz, Mexico) and cultured it in the laboratory on mixed prey of copepods and rotifers at a salinity of 20 g L^−1^. *C. caspia* is tolerant of a wide range of salinities. Most studies on *C. caspia* report its feeding and population growth on *Artemia* nauplii. Here we tested the effect of salinity on functional response and prey preference on *Brachionus plicatilis* (Rotifera), *Apocyclops panamensis* (Copepoda, Cyclopoida) and *Nitokra lacustris* (Copepoda, Harpacticoida). Experiments were conducted at 25°C. The hydrozoan killed several prey during functional response studies, which we assumed that it would eventually consume. On all prey test species, *C. caspia* showed a Type II functional response. Prey consumption, in terms of biomass, and preferences was higher on copepods than on rotifers. Prey consumption was higher at salinities of 10 and 20 g L^−1^ than of 30 g L^−1^.

## INTRODUCTION

Cnidarians are found in a range of salinities, from freshwater to marine ecosystems (Slobodkin and Bossert, 2010). *Cordylophora caspia* (Pallas, 1771) is a colonial euryhaline hydrozoan, first reported from the Ponto-Caspian basin (Anon, 2007). One or two species in this genus have been recorded (Dumont, 2009). Over the past century, it has spread widely on all the continents except Antarctica (CONABIO, 2015). The species was first observed in Brazil in 1858 but was officially recorded more than 100 years later (Deserti *et al.*, 2023). It has been reported several times in Mexico, but without any morphological data or images (Deserti *et al.*, 2023). It is widespread due to its capacity to tolerate a wide range of temperature and salinities, as well as its ability to produce dormant stage menonts (resting stages), which help it withstand adverse conditions (Folino-Rorem *et al.,* 2009). As most invasive species, it is reported to cause ecological damage, and also affects hydroelectric cooling systems from the widespread growth of colonies (Pucherelli *et al.,* 2018).

*C. caspia* is reported from freshwaters and saline systems (Deserti *et al.*, 2023). Although there are many reports, they are based only on observations in samples. In Mexico, all records are from estuaries and reefs in the Gulf of Mexico, except for one from a river in the State of Nuevo Leon (Deserti *et al.,* 2023). It has been suggested that the genus may represent a species complex (Folino-Rorem *et al.,* 2009). *C. caspia* tolerates wide salinity levels, while *Cordylophora lacustris* (Pallas, 1771) is found in freshwaters (Smith *et al.,* 2002).

Although not cultured often, a few studies indicate that *Cordylophora* feeds on several different prey taxa such as microcrustaceans, worms, mollusks and other invertebrates (Slobodkin and Bossert, 2010). Feeding experiments on *Artemia* sp. prey indicate that *C. caspia* recognizes its prey due to the presence of an amino acid, proline (Fulton, 1963). Studies on the feeding behavior of predatory animals, such as *Cordylophora*, are also important to estimate the impact of invasive taxa on their prey (Dick *et al.,* 2014). Feeding behavior is often analyzed based on numerical and functional responses, and prey preference studies. The long lifespan of *C. caspia* requires more time to study the numerical responses, but food consumption with increasing prey availability and choice of prey can be recorded easily. Such studies help predict the impact of predators on the prey population in their environment. Several biotic and abiotic factors influence prey consumption by aquatic predators (Nandini *et al.,* 2003; Cansse *et al.,* 2020). Under stressful conditions, prey consumption and prey preference may change considerably. For aquatic species, salinity is one of the major stress factors. When prey consumption studies are conducted under different salinities, the relative tolerance levels of prey species and the predator must be considered. The cyclopoid *Apocyclops panamensis* and the harpacticod *Nitokra lacustris* are euryhaline. Both these copepods have been cultured under different salinities from 6–40 g L^−1^ (Lindley *et al.,* 2011; Punnarak *et al.,* 2017). The rotifer *Brachionus plicatilis* is a species complex (Mills *et al.,* 2017). It is widely used as starter diet for larval fish and decapod crustaceans. Therefore, *B. plicatilis* has been cultured in a wide range of salinity levels from 5–25 g L^−1^ (Nandini *et al.,* 2019). All the prey species and the predator in this study tolerate a wide range of salinity levels, allowing us to quantify the prey consumption by *C. caspia* at different salinity levels.

A predator’s ability to capture prey items is also dependent on the quantity of prey offered. Most aquatic predators show increased prey consumption with increasing availability (functional response), until an asymptote is achieved, beyond which further prey availability does not increase the rate of consumption (Case, 1999). Most predators including freshwater cnidarians, such as *Hydra* also show this trend. However, in functional response evaluations, the killed prey items are consumed within the test period (Nandini *et al.,* 2003). In certain cases, predators continue to kill available prey, but consumption is delayed, i.e. kill rate is not proportional to consumption rate (Sheriff *et al.,* 2020). However, it can be assumed that killed prey items are eventually consumed. It is not known if *C. caspia* has this tendency.

In nature *C. caspia* encounters a great variety of prey items, such as rotifers, copepods and other invertebrates (Slobodkin and Bossert, 2010). However, prey selection by the predator depends on the relative densities of the former. Unfortunately, field-collected *C. caspia* is not suitable for gut content analyses as the animals shrink upon preservation and based on our preliminary observations, prey remnants are not always visible. Therefore, laboratory tests in which the predator is exposed to different prey items of known density are helpful to understand the prey preferences.

Here, we tested the functional response and prey preference of *C. caspia* offered three prey types: the cyclopoid *Apocyclops panamensis*, the harpacticoid copepod *Nitokra lacustris* and the rotifer *Brachionus plicatilis* at three test salinities of 10, 20 and 30 g/L. In our field collections from the river Tuxpan in the State of Veracruz, Mexico, we observed *A. panamensis* and *B. plicatilis*. However, we did not observe any zooplankton in the guts of the cnidarian predator. Our initial tests indicate that *C. caspia* can tolerate salinities from 5–40 g/L but does not survive in freshwater. We hypothesized that the prey consumption rate would decline at high prey densities and that the predator would consume similar prey numbers at all salinity levels due to its wide tolerance to salt concentration. We also hypothesized that *C. caspia* would have a greater preference and prey consumption for the smaller and slower prey such as the harpacticod (*N. lacustris*) and the rotifer (*B. plicatilis*) as compared to the evasive cyclopoid, *A. panamensis*.

## MATERIALS AND METHODS

We isolated three prey species, the cyclopoid *A. panamensis*, the harpacticoid *N. lacustris* and the rotifer *B. plicatilis* from a shrimp farm in Mazunte, Oaxaca State (Mexico) and cultured them for more than two years prior to use in the experiments. We used the green alga *Nannochloropsis oculata*, cultured on the F/2 Guillard medium as food for the zooplankton (Guillard, 1975). *C. caspia* was isolated from the Tuxpan River in State of Veracruz. Our personal observations show that the river had a salinity gradient from 5–25 g L^−1^ during an annual cycle. We used the above-mentioned zooplankton as prey for the predator *C. caspia*. All species were cultured at 20 g L^−1^; we used Instant Ocean Sea salt for the culture media.

We tested the salinity tolerance of *C. caspia* at 0, 5, 10, 15, 20, 30 and 40 g L^−1^. Each salinity level was replicated four times in separate 15 mL plastic containers at 25°C. In 10 mL medium at each salinity, we introduced one individual of *C. caspia*. After 24 h, we counted the number of predators remaining alive.

We conducted the functional response experiments in 15 mL plastic containers, with 10 ml of medium, at 25°C, which is around the mean temperature in the river. We tested the prey consumption at three salinities of 10, 20 and 30 g L^−1^. Adult copepods (males and non-ovigerous females) were introduced into each recipient at densities of 0.5, 1, 1.5, 2, 3, 4 and 6 individuals ml^−1^. Rotifers were introduced at densities of 1,2, 4,6, 8 and 12 individuals ml^−1^. Before introducing the prey, we introduced one individual of *C. caspia*, around one week old, into each container. Then we introduced the prey of each species at the desired density into each recipient. The predators were allowed to feed for 2 h, after which we counted the number of living prey in the test jars. The difference between the original and final density was considered as the number of prey killed. While many more prey were killed, we observed that these zooplankton were not consumed entirely during the study period. In such cases, the soft parts of the prey were sucked out and the lorica of the rotifers or the exoskeleton of the copepods were discarded.

A type II functional response curve was fitted to each treatment set of prey consumption over prey density using the Michelis—Menten formula (Nandini and Sarma, 1999)


$$ Y= aX/\left(b+X\right), $$

where Y is the expected prey consumption per predator at a given prey density, *a* is the maximum number of prey consumed, X is the prey density in the medium and b is the prey consumed at half of *a*.

Tests of prey preference were conducted with the same experimental setup, except that we introduced 20 individuals of each copepod species and 50 rotifers into each recipient; we set up 5 recipients at each salinity. After 2 h of feeding, we counted the living prey left and the difference indicated the number of prey killed. More prey were killed than actually consumed. We also calculated the biomass of prey consumed for each species. The biomass was calculated by measuring the length and width of 10–20 non-gravid female prey individuals. Based on the following formulae (where W is weight of females in μg and L is the length in mm) we calculated the biomass of each prey type and then, based on the numbers consumed, the total prey biomass (Table 1).

**Table I:** Data on the body length, width and biomass of the prey species (without eggs). Shown are mean ± SE based on the data from the number of individuals shown in the table.

Species	Formula from	Length (μm)	Width (μm)	Weight (μg)	Individuals measured
*Apocyclops panamensis*	Bottrell *et al.,* 1976	701 ± 15	236 ± 6	1.1 ± 0.05	10
*Nitokra lacustris*	Goodman, 1980	464 ± 7	133 ± 3	0.82 ± 0.1	9
*Brachionus plicatilis*	Dumont *et al.,* 1975	180 ± 5	148 ± 4	0.112 ± 0.0009	18

*A. panamensis* (cyclopoid dry weight): ln(W) = 1.9526 +  2.3990 × ln(L) (Bottrell *et al.*, 1976).

*N. lacustris* (harpacticoid) dry weight: W = 14.01 L—5.69, (Goodman, 1980).

*B. plicatilis* W = 8.83 × 10^−5^ L^1.44^, (Dumont *et al.,* 1975).

In the last case, the original formula was derived for *Brachionus calyciflorus*. However, the results obtained are similar to the dry weight reported by Theilacker and McMaster (1971) for *B. plicatilis*.

We compared differences in the maximum prey consumption (*a*) in terms of numbers and biomass, using two-way Analysis of Variance (ANOVA). Prey preference was calculated using Manly’s α (Manly, 1974).

## RESULTS

**Fig. 1:**
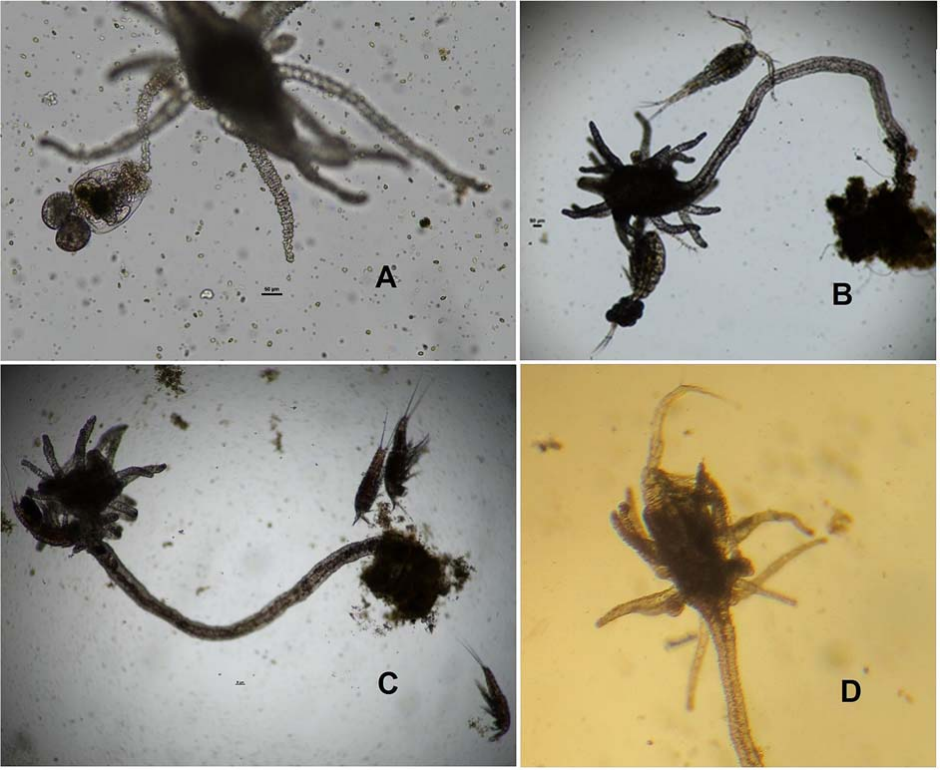
*Cordylophora caspia* feeding on *Brachionus plicatilis*  **(a)**, *Apocyclops panamensis*  **(b)**, *Nitokra lacustris*  **(c)** and an *Apocyclops* in the cnidarian’s gastrovascular cavity **(d)**.

Our observations indicated that *C. caspia* was a surplus killer; it killed many more prey than it could consume within the experimental duration. They killed prey in the vicinity, either accidentally or on purpose, and to presumably consume them later (Fig. 1a–c). While many prey were killed in the medium, only 3–4 copepods were close to the stolon of the predator. In a few cases, the predator consumed the prey and egested the remains. In such cases, depending on the hunger level, the complete prey was in the gut within a few minutes of capture (Fig. 1d). We also observed some body parts, such as eye spots, within the slurry in the gut. For both copepod species, irrespective of prey density, more than 80% of prey were killed during the observation period. However, in the case of the smaller prey (rotifers < 200 μm) less than 40% of the items were killed during the test period.

**Fig. 2:**
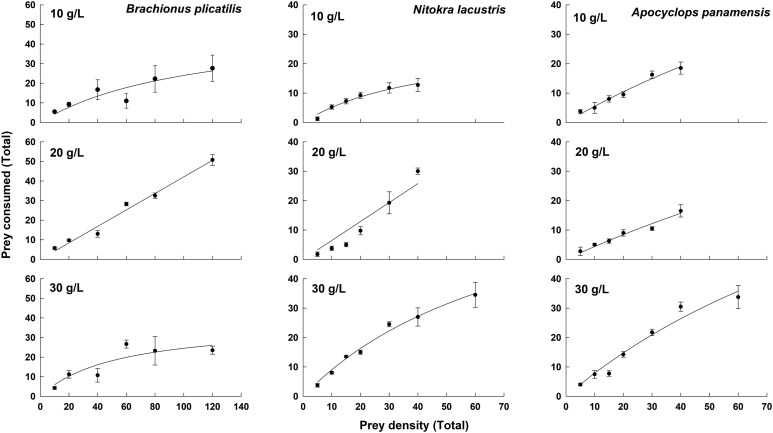
Functional response curves of *Cordylophora caspia* in relation to different densities of zooplankton prey *Brachionus plicatilis* (Rotifera), *Apocyclops panamensis* (Cyclopoida) and *Nitokra lacustris* (Harpacticoida). Data represent mean ± SE based on four replicates for each prey species and density.

Predator consumption (individuals eaten or killed) of *B. plicatilis*, *A. panamensis* or *N. lacustris* increased with increasing prey availability (Fig. 2). An asymptote in relation to increasing prey availability was observed at the highest salinity tested of 30 g L^−1^. This asymptote was often not observed at 10 or 20 g L^−1^.

**Fig. 3:**
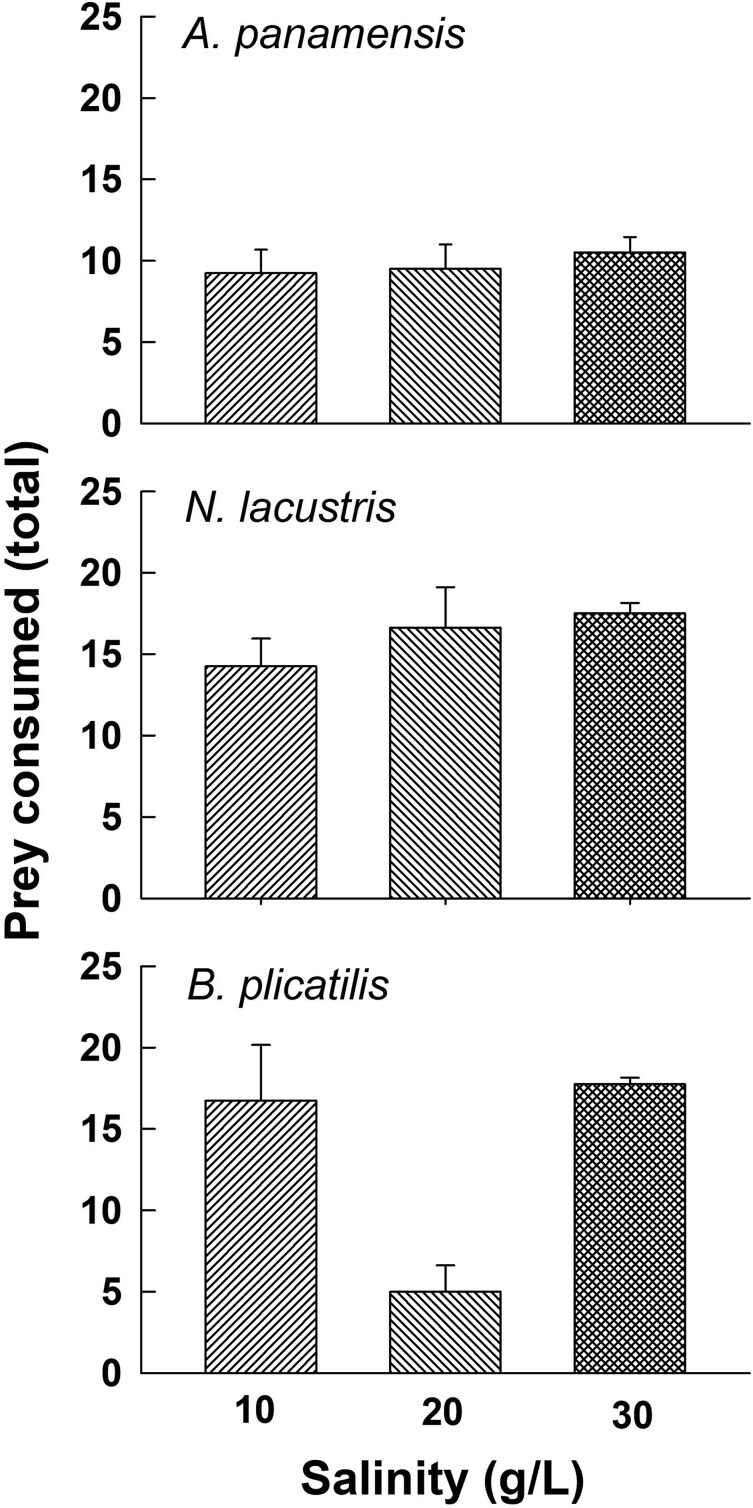
Zooplankton prey (*Brachionus plicatilis, Apocyclops panamensis* and *Nitokra lacustris*) (numbers) consumption by *Cordylophora caspia* at three salinities of 10, 20 and 30 g L^−1^. Data represent mean ± SE based on four replicates for each prey species and density.

**Fig. 4:**
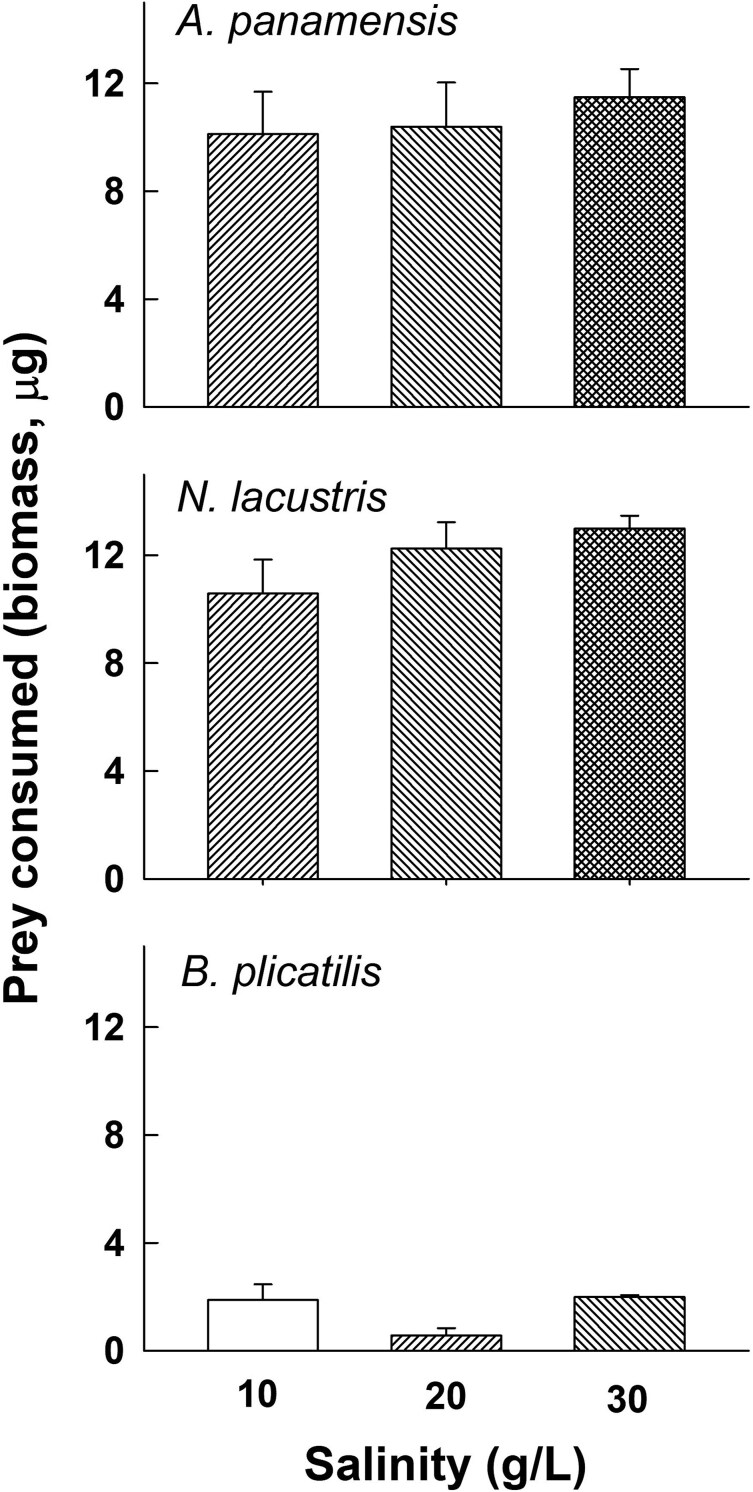
Zooplankton prey (*Brachionus plicatilis, Apocyclops panamensis* and *Nitokra lacustris*) (biomass) consumption by *Cordylophora caspia* at three salinities of 10, 20 and 30 g L^−1^. Data represent mean ± SE based on four replicates for each prey species and density.

There was a significant increase (P < 0.05, two-way ANOVA, Table 2) in prey species consumption (*B. plicatilis, N. lacustris* or *A. panamensis*), with regard to prey density or type (species), with increasing salinity (Fig. 3). Prey type consumption was also significantly affected by salinity. In addition, there was a significant interaction of prey type and salinity on consumption (p < 0.05, two-way ANOVA, Table 2). Regarding prey consumption in terms of biomass (Fig. 4), there was a significantly greater biomass of copepods consumed than rotifers (P < 0.001, two-way ANOVA, Table 2) with no significant further effects (main or interaction) of salinity (P > 0.05).

**Table II:** Two-way ANOVA conducted on the prey consumption (*Brachionus plicatilis*, *Apocyclops panamensis* and *Nitokra lacustris*, as numbers or biomass) by the predatory *Cordylophora caspia* in relation to salinity.

Source of Variation	DF	SS	MS	F	P
*Prey numbers*					
Salinity	2	148.167	74.083	3.781	0.036
Prey	2	241.167	120.583	6.155	0.006
Salinity × prey interaction	4	279.667	69.917	3.569	0.018
Residual	27	529.000	19.593		
*Prey biomass*					
Salinity	2	12.931	6.465	1.432	0.256
Prey	2	915.387	457.693	101.407	<0.001
Salinity × prey interaction	4	11.254	2.813	0.623	0.650
Residual	27	121.862	4.513		

DF: Degrees of freedom; SS: Sum of squares; MS: Mean Square; F: Fisher's ratio; P: Probability

**Fig. 5:**
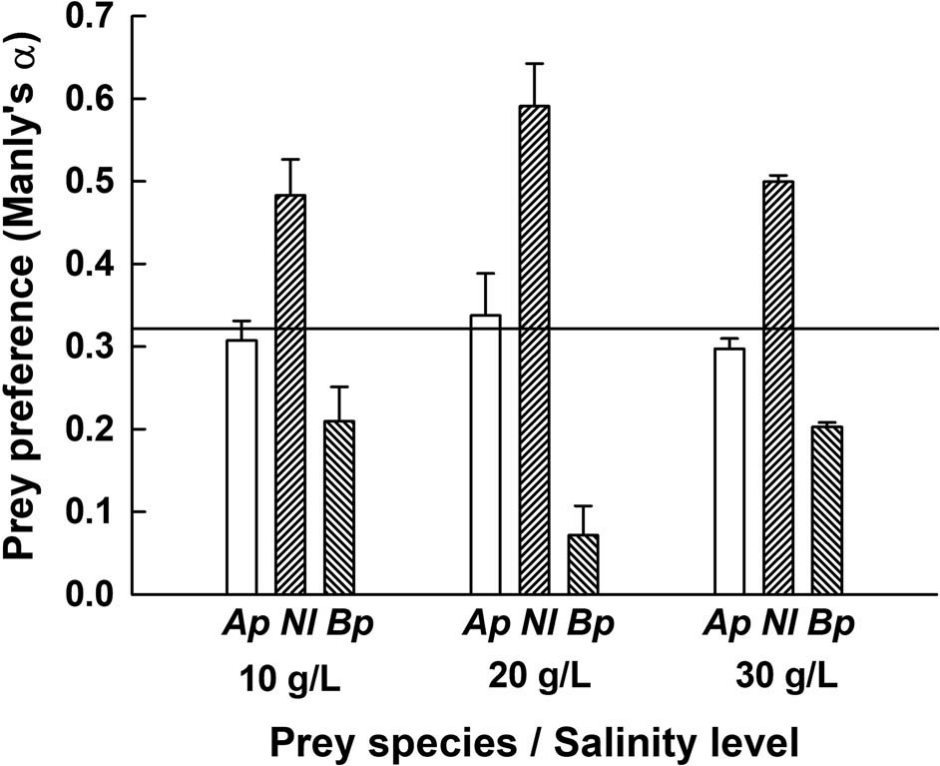
Prey preference (Manly’s α) by *Cordylophora caspia* fed mixed prey (*Brachionus plicatilis, Apocyclops panamensis* and *Nitokra lacustris*). Each data bar represents mean ± SE based on four replicates. Manly’s α values above the horizontal line represents positive selectivity while values below the line represents prey consumption in relation to availability.

Prey preferences (Manly’s *α*) did not change with increasing salinity (Fig. 5). Based on the number of prey killed, *C. caspia* always preferred the harpacticoid *Nitokra* over the cyclopoid *Apocyclops*. *Brachionus* was least preferred.

## DISCUSSION

The salinity of the river Tuxpan ranged from 0–25 g L^−1^ depending on the site. During a previous study on zooplankton diversity in the river (Nandini and Sarma, *unpublished data*), we sporadically found *C. caspia* at different sites, but always at salinities > 5 g L^−1^. Our laboratory observations also indicate that the species we have isolated from this river does not survive in freshwater. However, it is known to be a euryhaline species capable of tolerating a wide range of salinities from 5–40 g/L. Fulton (1963) indicates that *C. caspia* is a euryhaline, but saline species; its nematocysts cease to function in freshwater. A study by Folino-Rorem and Renken (2018) also indicates that this may be a euryhaline species or one represented by freshwater and salinity tolerant clades. Further research on the molecular taxonomy of the species is warranted.

The feeding behavior of *Cordylophora* was different from several other invertebrate predators. Fulton (1963) showed that *Cordylophora* detects proteases from the prey. Some studies show that this cnidarian ingests organisms such as mollusk larvae (Pucherelli *et al.,* 2018); we also observed that *Cordylophora* ingested each prey individually. We have observed a slurry of the prey and its egested exoskeleton. While the predator was simultaneously handling a couple of prey individuals with its tentacles, it also had 3–4 dead copepods around it.

Functional response studies normally consider the prey eaten by predators for the analyses of prey consumption in relation to prey availability (Holling, 1959). In this study, we observed that the predator indulged in surplus killing, i.e. killing more prey than consumed within the study period. Most studies on the subject of surplus killing have been conducted on mammalian predators; Chopin-Rodríguez *et al.* (2022) suggest that this is a rare behavior, common under conditions of unpredictable prey availability. It has been suggested that surplus killing reduces competition from conspecific predators (Lounibos *et al.,* 2008). It remains to be seen whether there exists any competition for prey killed by cnidarian predators in their natural habitat. Thus, while *Cordylophora* kills more prey, it handles only 2–3 individuals at a time.

If the number of prey killed is considered, as we did here, it appears that the predator shows a Type I or Type II functional response depending on the prey type and salinity. We observed a Type I response at densities of 12 individuals ml^−1^ of rotifers and 4 individuals ml^−1^ of copepods, densities far greater than those found in natural systems. When 6 individuals ml^−1^ of copepods were offered, an asymptote in relation to prey killed was evident (Type II). Similar trends have been reported in feeding studies on the coral, *Desmophyllum dianthus* (Höfer *et al.,* 2018) and the jellyfish, *Aurelia aurita* (Yurtseva *et al.,* 2018).

The maximum number of prey consumed did not differ with salinity for copepods or rotifers. This is also in accordance with our observations on the mortality of *Cordylophora* across the range of salinities from 0–40 g L^−1^. Over the 24 h period, mortality was observed only at 0 g L^−1^. Although studies are being conducted on *C. caspia* from the early 1960s (Fulton, 1963), we found no study indicating the number of prey eaten. However, the number of prey consumed is similar to that reported for the related freshwater *Hydra* (Rivera-De la Parra *et al.,* 2016).

In terms of densities, we observed that the predator ate similar numbers of copepods (cyclopoids or harpacticoids) but more individuals of the rotifer; however, in terms of biomass, the consumption was higher on copepods than on rotifers. Biomass consumption was also greater on *Nitokra* than *Apocyclops,* presumably because of the lower swimming speed and frequent periods of rest of the former as compared to the latter (Williamson and Reid, 2001; Fechter *et al.,* 2004). It appears that the small size of rotifers is an effective defense against this predator, as has been shown for other invertebrate predators offered small sized rotifer prey (Sarma and Nandini, 2007). Yurtseva *et al.* (2018) have also shown that the jellyfish, *A. aurita* consumes fewer rotifers compared to larger prey such as copepods.

*C. caspia* is a biofouling organism and probably damages fish and shrimp larvae in aquaculture practices. Published works indicate that invasive species can be beneficial or harmful to other invasive species (Sax *et al.,* 2022). For example, Matern and Brown (2005) report that the Asian shimifouri goby (*Tridentiger bifasciatus*), an invasive in Californian estuaries, feeds on *C. caspia*. Further studies are required to test biological and physical control of *Cordylophora* to prevent damages to human-made structures and fragile ecosystems (Wintzer *et al.,* 2011). The site from where we isolated *C. caspia*, the river Tuxpan, is extensively used by local communities for harvesting the mullet (*Mugil liza*) and some species of crabs and shrimps. It is quite possible that *C. caspia* may have a significant adverse effect of the early developmental stages of these fish and crustacean species.

## CONCLUSION

Our findings indicate that *C. caspia* consumed more copepods than rotifers. Between the two prey species of copepods, it preferred and consumed the harpacticoid *N. lacustris* in greater numbers than the cyclopoid *A. panamensis*. Salinities ranging from 10 to 30 g L^−1^ had no effect on the predator’s feeding behavior. The tendency of the predator to indulge in surplus killing indicates that it may have a significant adverse impact on zooplankton communities in nature.

## Data Availability

Data will be made available on reasonable request.
